# Saving the crossing suture during endoscopic sleeve gastroplasty

**DOI:** 10.1016/j.vgie.2024.06.013

**Published:** 2024-07-05

**Authors:** Mehmet Celikbilek, Manoel Galvao Neto

**Affiliations:** 1 MC Gastroenterology Clinic, Ankara, Turkey; 2 Mohak Bariatrics and Robotics Center, Sri Aurobindo University, Indore, India; 3 Orlando Health Weight Loss and Bariatric Surgery Institute, Orlando, Florida, USA; 4 Endovitta Institute, Sao Paulo, Brazil

## Case presentation

Endoscopic sleeve gastroplasty (ESG) is a gastric restrictive procedure that is increasingly being performed to treat obesity. The series of transmural sutures are applied to remodeling of the greater gastric curvature, resulting in both volume reduction and foreshortening. This fully-endoscopic procedure has many desirable advantages over laparoscopic operations, including a truly scarless technique, shorter length of hospital stay, and improved perioperative outcomes. In adults with overweight or obesity, the American Society for Gastrointestinal Endoscopy-European Society of Gastrointestinal Endoscopy^1^ suggests the use of gastric remodeling with lifestyle modification. ESG is suggested for patients with a body mass index (BMI) ≥30 kg/m^2^ with or without an obesity-related comorbidity or a BMI of 27 kg/m^2^ to 29.9 kg/m^2^ with at least 1 obesity-related comorbidity.^1^^,^^2^

**Figure 1:**
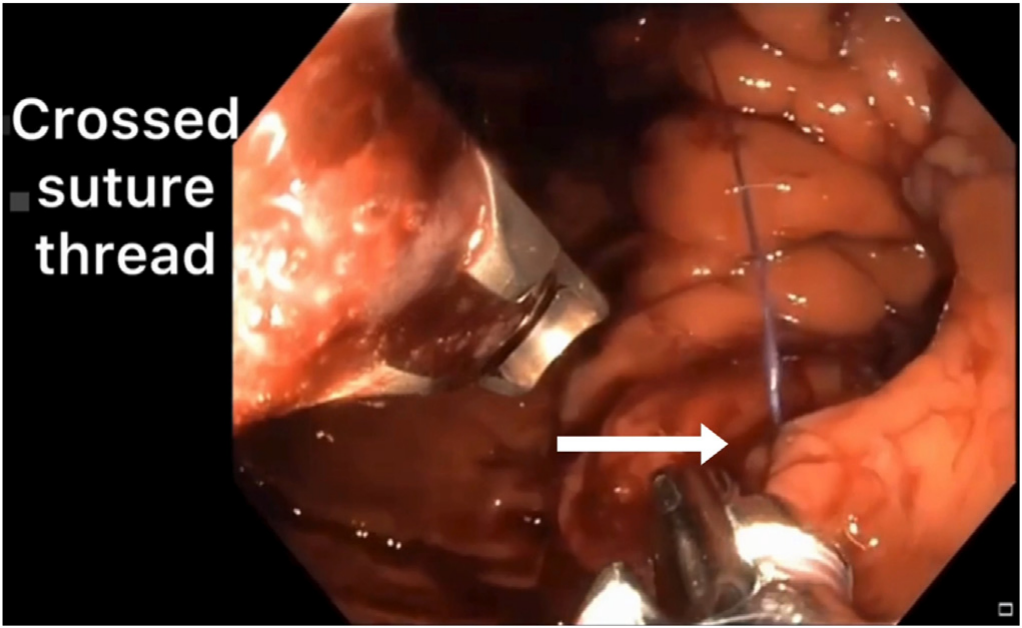
Shown is the crossed and trapped suture line on the posterior wall of the stomach.

A 46-year-old woman underwent ESG for the treatment of obesity using an endoscopic suturing device (OverStitch; Apollo Endosurgery, Austin, Tex, USA) attached to a double-channel adult gastroscope, Olympus CV 190 (Olympus Medical Systems Corp, Tokyo, Japan) and insufflation with carbon dioxide. During the bite for the base of the second U-shaped suture pattern on the posterior wall, the suture thread crossed over and made it impossible to cinch (Fig. 1). First, we continued and made the final bite to finish the suture line on the posterior wall. We tried to catch the thread and pull it back, but it failed. Then, we caught the thread with the second forceps and pulled it back. While pulling the thread back through the left channel of the endoscope, we simultaneously pushed and held the distal part of the thread through the right channel of the endoscope with the other forceps. When we had saved the thread from crossing, we cinched and finished the second suture line as a straight line (Video 1, available online at www.videogiejournal.org).

The U-shape suture pattern was essentially developed to be straighter and avoid suture crossing. Despite this, suture crossing may occur along the posterior wall because of maneuvers. The most common cause of suture crossing is closing the needle on the right side of the loop thread when charging the needle. To avoid suture crossing, there should be no thread between the 2 towers of overstitch device during bite. Because of their high cost, it is very important not to waste sutures. In this article, we have explained how to save the crossed thread during ESG with a 2-forceps technique.

## Disclosure

Prof Neto is an international consultant for Boston Scientific. Dr Celikbilek disclosed no financial relationships.

## Supplementary data

Video 1 Shown is thread crossing and the 2-forceps technique to save the crossed thread during ESG.
